# Conformal Coverage of ZnO Nanowire Arrays by ZnMnO_3_: Room‐temperature Photodeposition from Aqueous Solution

**DOI:** 10.1002/cphc.202300250

**Published:** 2023-09-22

**Authors:** Karin Rettenmaier, Gregor A. Zickler, Thomas Berger

**Affiliations:** ^1^ Department of Chemistry and Physics of Materials University of Salzburg Jakob-Haringer-Straße 2a 5020 Salzburg Austria

**Keywords:** green chemistry, nanowire array, nanocomposite, electrodeposition, pseudocapacitance

## Abstract

Compositionally and structurally complex semiconductor oxide nanostructures gain importance in many energy‐related applications. Simple and robust synthesis routes ideally complying with the principles of modern green chemistry are therefore urgently needed. Here we report on the one‐step, room‐temperature synthesis of a crystalline–amorphous biphasic ternary metal oxide at the ZnO surface using aqueous precursor solutions. More specifically, conformal and porous ZnMnO_3_ shells are photodeposited from KMnO_4_ solution onto immobilized ZnO nanowires acting not only as the substrate but also as the Zn precursor. This water‐based, low temperature process yields ZnMnO_3_/ZnO composite electrodes featuring in 1 M Na_2_SO_4_ aqueous solution capacitance values of 80–160 F g^−1^ (as referred to the total mass of the porous film i. e. the electroactive ZnMnO_3_ phase and the ZnO nanowire array). Our results highlight the suitability of photodeposition as a simple and green route towards complex functional materials.

## Graphical abstract

**Crystalline–amorphous biphasic ZnMnO_3_** was photodeposited by a one‐step, room‐temperature process from an aqueous precursor solution onto ZnO nanowires. The specific morphology of the photodeposit and its homogeneous dispersion at the nanowire surface give rise to an electrode architecture, where ZnMnO_3_ shells act as an electroactive, pseudocapacitive phase in aqueous electrolytes.

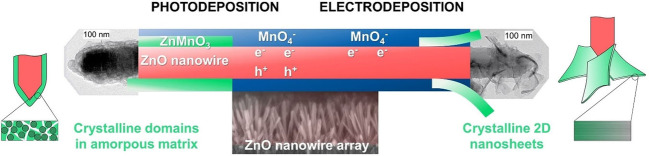

## Introduction

The aim of combining high performance with economic and environmental sustainability has triggered extensive research on binary oxides based on earth‐abundant and cheap transition metals such as manganese or iron as active materials in batteries and supercapacitors.[^1^, ^2^, ^3^, ^4^, ^5^, ^6^] The availability of environmentally benign synthesis routes^[4]^ together with the possibility of using metal oxide electrodes in aqueous electrolytes open up prospects for the development of green energy storage technologies. However, binary oxides often suffer from some shortcomings such as inherently low ionic and/or electronic conductivities, low electrocatalytic activity or limited long‐term stability, which hamper their broad application.[^1^, ^2^, ^3^] Mixed transition metal oxides allow to overcome some of these challenges and have recently emerged as promising electrode materials for ion batteries, metal‐air batteries and supercapacitors.[^7^, ^8^, ^9^, ^10^, ^11^] Correspondingly, there is an urgent need for simple and robust synthesis routes, which should ideally comply with the principles of modern green chemistry (e. g. by relying on water‐based low temperature processes).[^12^, ^13^] Photodeposition and electrodeposition have proven very useful for the synthesis of functional nanomaterials.[^14^, ^15^, ^16^, ^17^] Photodeposition, for instance, has been used extensively for the deposition of catalytic materials, such as metal and binary metal oxides, onto photocatalysts[^14^, ^18^, ^19^] and allows under appropriate conditions for a conformal coating of semiconducting nanostructures.^[20]^ In some cases, even more complex mixed oxide nanoparticles have successfully been deposited onto semiconductors using direct photodeposition from complex precursor solutions[^21^, ^22^, ^23^] or sequential photodeposition.^[23]^

Spinel‐type manganates constitute promising electrode materials for applications in electrochemical energy conversion and storage including electrocatalysis, pseudocapacitors and batteries.^[24]^ In these materials manganese ions may feature various oxidation states imparting to the structure a high density of active sites as well as a high electrical conductivity.^[24]^ On the other hand, the spinel‐type crystal structure features a high density of unoccupied octahedral sites, which together with tetrahedral sites form a three‐dimensional interconnected network leading to a unique diffusion topology in the crystal framework.^[25]^ Correspondingly, oxides with spinel structure have also attracted attention as potential cathode materials for multivalent ion batteries.^[26]^ In cation‐defective ZnMn_2_O_4_, for instance, reversible Zn^2+^ intercalation from aqueous media has been observed.^[27]^ In addition to tetragonal ZnMn_2_O_4_, ZnMnO_3_ represents a second type of zinc manganese oxide spinel. Application of ZnMnO_3_ as electroactive material is limited so far despite its promising electrochemical properties. Importantly, this defective cubic spinel is particularly difficult to synthesize via low temperature wet chemistry routes.[^28^, ^29^] In a very recent review,^[24]^ an ongoing need for the development of synthesis routes towards spinel‐type manganates featuring well‐defined and tunable properties has been identified. Specifically, optimization of the materials’ functional properties requires the minute control of their morphology, crystal structure, crystallinity, type and concentration of defects as well as the preparation of nanocomposites and heterostructures (including biphasic crystalline‐amorphous structures^[30]^).

Here we report on the room‐temperature, one‐step photodeposition of ZnMnO_3_ shells onto ZnO nanowires from an aqueous KMnO_4_ solution. The morphological and structural properties of the resulting nanocomposite films have been characterized by electron microscopy. The photodeposited shell covering single ZnO nanowires consists of nanocrystalline ZnMnO_3_ domains embedded in an amorphous ZnMnO_3_ matrix. This specific structure gives rise to an increased capacitance of the electrodes in aqueous electrolyte as compared to composites consisting of crystalline ZnMnO_3_ nanosheets immobilized on ZnO nanowire arrays.^[31]^ The results highlight the great potential of photodeposition as a simple and green synthesis strategy towards electroactive materials.

## Experimental Section

### Photodeposition of ZnMnO_3_

ZnO nanowire arrays electrodeposited on AZO (aluminum‐doped zinc oxide, ZnO : Al) and FTO (fluorine‐doped tin oxide, SnO_2_ : F)–coated glass slides (see Supporting Information) were used as substrates for the photodeposition of ZnMnO_3_. Photodeposition was performed in an argon (Ar 5.0)–purged 0.175 mM KMnO_4_ aqueous solution (pH 5, *V*=35 mL). For this purpose, the semiconductor layer was exposed from the substrate side (AZO/FTO–covered glass, Figure S1) to polychromatic UV/Vis light provided by a 1000 W Xe‐discharge arc lamp (LOT Quantum design), which was equipped with a water filter for IR removal. An irradiance *P*=330 mW cm^‐2^ reached the ZnO nanowire array after passing the AZO/FTO–covered glass as determined with a bolometer (International Light, IL1400 A). The photodeposition potential was measured at open circuit against an Ag/AgCl (1 M KCl) reference electrode (PalmSens). The photodeposition time ranged between 15 min and 120 min. After photodeposition the electrode was rinsed with ultrapure water and dried at room temperature in air.

### Electrodeposition of ZnMnO_3_

Electrodeposition of ZnMnO_3_ was performed in a three–electrode cell. A ZnO nanowire electrode was used as the working electrode, a flat Pt spiral aligned parallel to the working electrode acted as the counter electrode and an Ag/AgCl (1 M KCl) electrode (PalmSens) was used as the reference electrode. A nitrogen (N_2_ 5.0)–purged 0.175 mM KMnO_4_ (Sigma Aldrich, purity≥99 %) aqueous solution (*V*=80 mL) was used as the precursor for ZnMnO_3_ deposition. In a previous study, electrodeposition was performed in pure 0.175 mM KMnO_4_ aqueous solution (pH 5.3±0.2) at a deposition potential *E*_Ag/AgCl_=0.000 V.^[31]^ In this study, we adjusted the pH value of the precursor solution (to pH 4, pH 7 and pH 10, respectively) by adding KOH (Merck, EMSURE® for analysis) or HCl (Merck, 32 %). We adjusted the electrodeposition potential by ±0.059 V per pH unit (i. e. *E*_Ag/AgCl_=0.033 V, −0.144 V and −0.321 V at pH 4, pH 7 and pH 10, respectively) to account for the pH‐dependent band alignment of metal oxide semiconductors. The electrodeposition time was set to 45 min in each case. The total charge passed during electrodeposition depends slightly on the pH value and accounts for 330±70 C g^−1^ at pH 4, 280±50 C g^−1^ at pH 7 and 470±60 C g^−1^ at pH 4.

### Electrochemical Characterization

Electrochemical characterization of ZnO and ZnMnO_3_/ZnO electrodes was carried out with a computer–controlled Autolab PGSTAT302 N potentiostat (Metrohm). Measurements were performed in a three‐electrode cell using a platinum wire as the counter electrode and an Ag/AgCl (3 M KCl) reference electrode (BasInc). A N_2_–purged 1 M Na_2_SO_4_ aqueous solution was used as the electrolyte. Cyclic voltammograms were recorded at various scan rates (0.005 V s^−1^≤*v*≤0.100 V s^−1^) in a potential window −0.1 V≤*E*≤1.0 V. Galvanostatic charging and discharging was performed for constant current densities ranging between 0.075 A g^−1^ and 8.0 A g^−1^ in a potential window −0.1 V≤*E*≤1.0 V. For the sake of comparability, open circuit potentials measured upon photodeposition of ZnMnO_3_ as well as potentials measured/applied upon electrochemical characterization are referred to an Ag/AgCl (3 M KCl) reference electrode throughout the paper and will be indicated as *E*_Ag/AgCl_.

### Spectroscopic and Microscopic Sample Characterization

Scanning electron micrographs were recorded on a Zeiss Gemini Ultra 55 scanning electron microscope (SEM), which is equipped with a field emission gun, using an in‐lens secondary electron detector.

A JEOL F200 (scanning) transmission electron microscope (STEM/TEM) equipped with a cold field emission gun and operated at 200 kV was employed to record (high resolution) transmission electron micrographs on a TVIPS F216 CMOS camera (2k x 2k). For details on sample preparation for TEM analysis, see the Supporting Information.

A windowless JEOL Centurio energy‐dispersive X‐ray detector (100 mm^2^, 0.97 srad, energy resolution <133 eV) contained within the transmission electron microscope was used for energy‐dispersive X‐ray (EDX) spectroscopic analysis. For details, see Ref. [31].

UV/Vis spectra of the immobilized films were recorded with a PerkinElmer LAMBDA 1050 UV/Vis/NIR spectrophotometer equipped with a 150 mm integrating sphere. Diffuse reflectance (R) data were converted into the Kubelka‐Munk function, F(R), which is represented in the spectra as a function of wavelength.

## Results and Discussion

### Morphology, Composition and Crystal Structure of Composite Films

**Figure 1:**
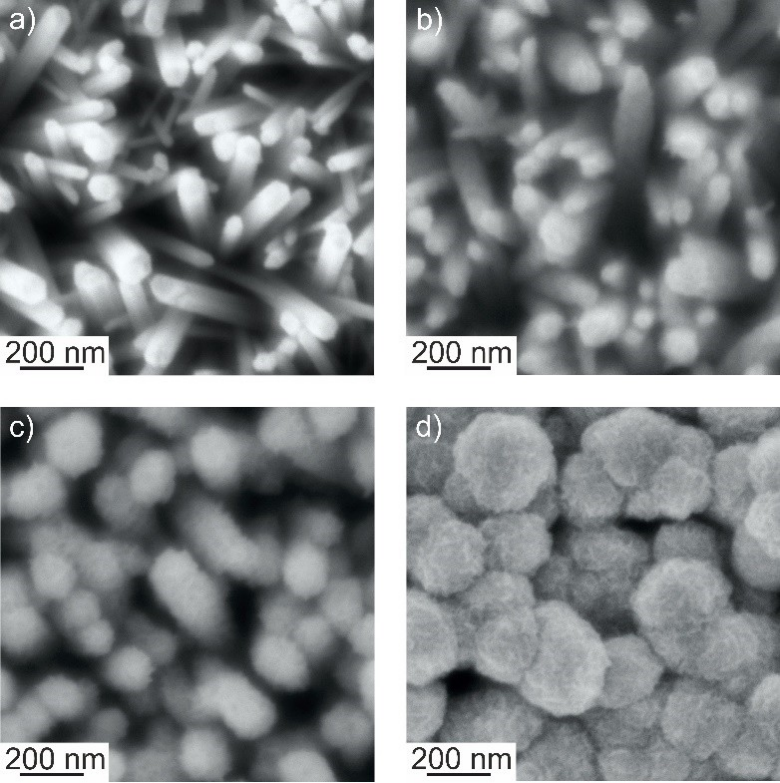
Top view scanning electron micrographs of a ZnO nanowire electrode before photodeposition (a) and of ZnMnO_3_/ZnO composite electrodes after 30 min (b), 45 min (c), and 120 min (d) of photodeposition.

**Figure 2:**
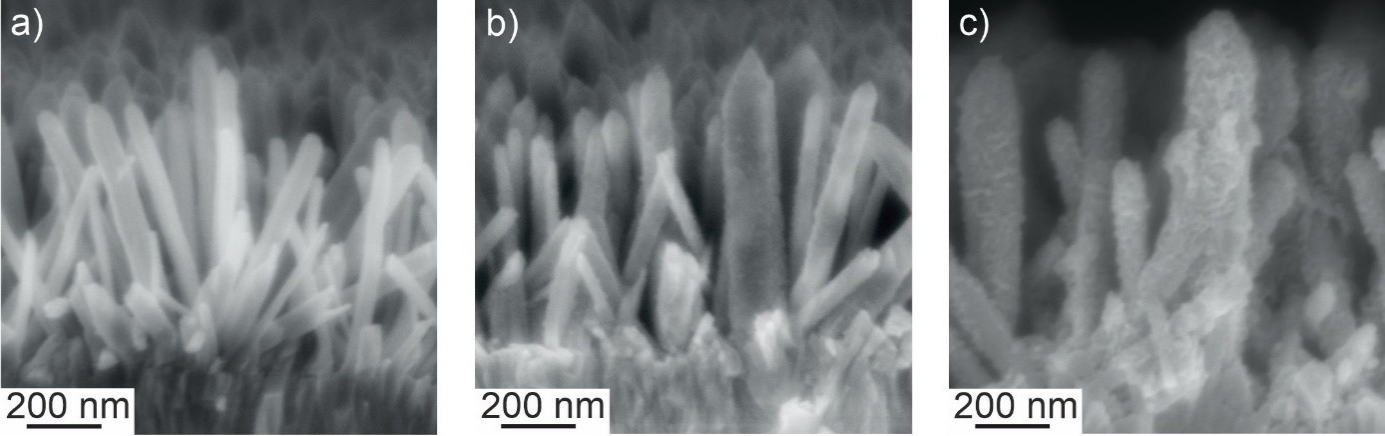
Cross section scanning electron micrographs of a ZnO nanowire electrode before photodeposition (a) and of ZnMnO_3_/ZnO composite electrodes after 30 min (b) and 45 min (c) of photodeposition.

Electrodeposited ZnO nanowires (Figures 1a and 2a) are hexagonal prisms with an average length of 700±200 nm and a diameter of 50±25 nm^[31]^ and feature the hexagonal wurtzite structure.^[31]^ Nanowires are elongated along the [0001] direction and are single crystals.^[31]^ Nanowire growth along the c‐axis was attributed in previous studies to the preferential adsorption of negatively charged Cl^−^ ions on high surface energy (0001) facets.[^32^, ^33^]

Top view as well as cross section scanning electron micrographs of nanowire films were acquired before and after photodeposition. For photodeposition times≥30 min, top view SEM images clearly reveal the appearance of a new phase at least in the upper part of ZnO nanowires (Figure 1). After 45 min of photodeposition, significantly increased nanowire diameters are discernable. However, individual nanowires are still separated one from the other thus preserving the porous structure of the nanowire array. However, after 120 min of photodeposition the nanowire diameter has increased to an extent, which leads to the merging of neighboring nanowires. Clearly, the newly formed sponge‐like phase fills the voids between nanowires upon prolonged photodeposition. For photodeposition times≤45 min cross section SEM images point to a homogeneous and conformal coverage of the entire nanowires by the newly formed phase (Figure 2b and c).

**Figure 3:**
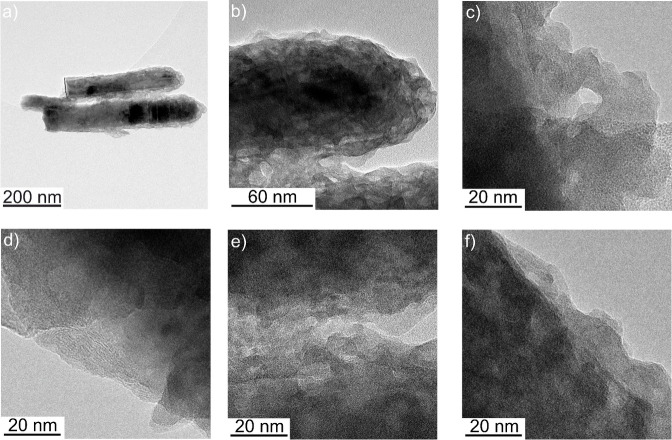
High resolution transmission electron micrographs of ZnMnO_3_/ZnO composite nanowires (photodeposition time: 45 min).

High resolution (HR) TEM images further corroborate deposit formation at the nanowire surface (Figures 3 and S2). The newly formed phase at the nanowires’ lateral and top regions features a homogeneous thickness and a porous morphology whereas the nanowire core seems to remain intact. However, the buried interface between the ZnO nanowire core and the newly formed shell is not clearly discernable. HRTEM images of the photodeposit reveal the presence of nanocrystalline domains (<10 nm) embedded in an amorphous matrix. Diffraction spots originating from these crystalline domains are visible in the Fast Fourier Transformation (FFT) patterns (Figure S2) and the corresponding lattice spacings are listed in Table S1. The small size of crystalline domains together with the low amount of the newly formed phase may explain, why the photodeposits are silent in X‐ray diffraction (not shown).

**Figure 4:**
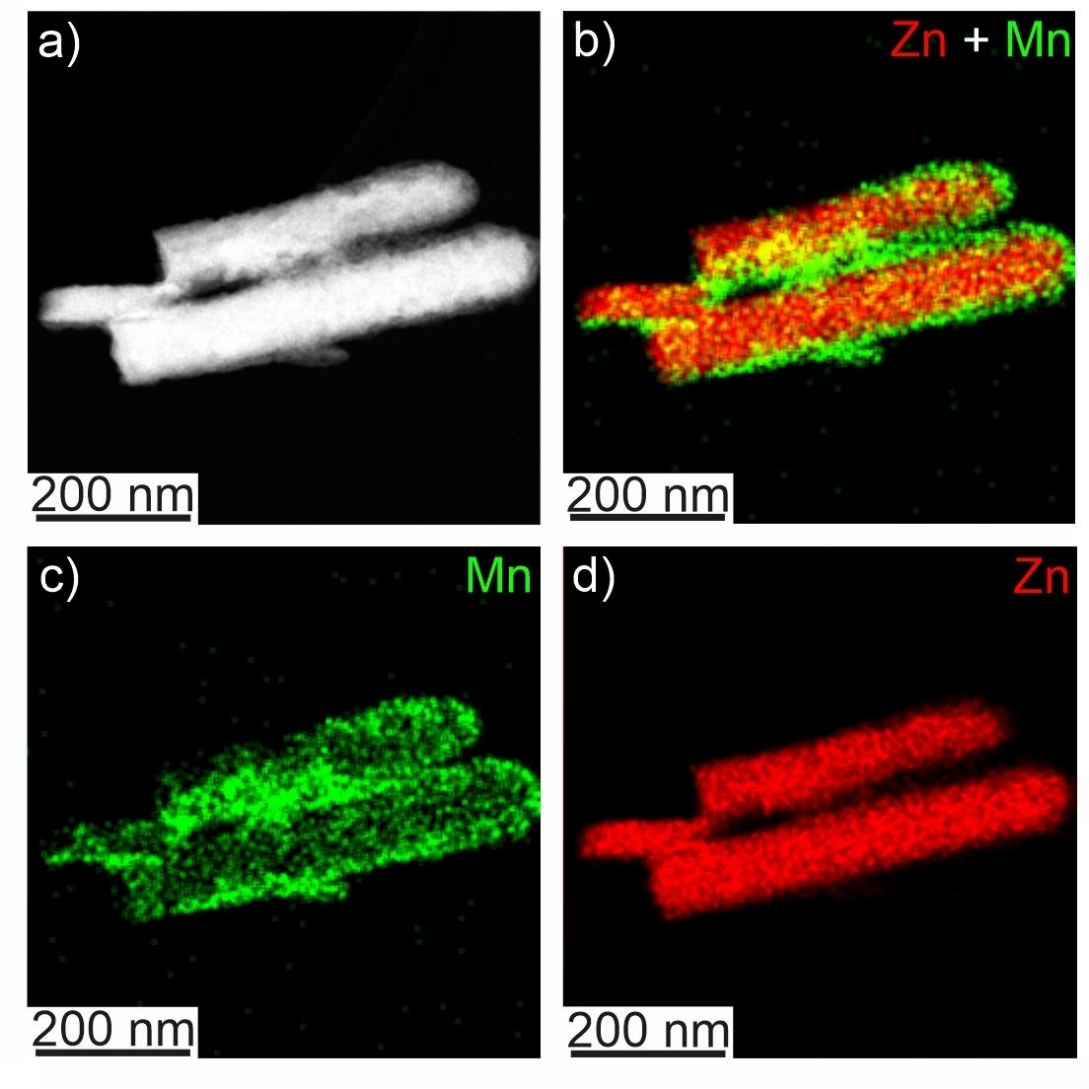
STEM‐HAADF image (a) and elemental intensity maps as obtained by EDX analysis of composite nanowires (b–d). Single elemental intensity maps of Mn (c) and Zn (d) are combined to a mixed elemental map (b).

The elemental composition of the photodeposit was investigated by EDX analysis. The core‐shell structure of the nanowires is clearly visible in EDX intensity maps (Figure 4), which confirms the presence of Mn and Zn in the photodeposited phase. The new phase is distributed over the entire surface of ZnO nanowires (Figure 4c and d). The shell is also visible in the STEM high angle annular dark field (HAADF) image (Figure 4a) due to the contrast difference resulting from very different densities of the two phases (i. e. the porous photodeposit shell and the dense ZnO core). The quantification of Mn and Zn present in the photodeposited phase via EDX yields an approximate Mn to Zn atomic ratio of 1.22 : 1.00. The elemental ratio of Mn : Zn of ~1 : 1 and the lattice spacings deduced from FFT patterns (Table S1) point to the photodeposition of nanocrystalline domains consisting of the defective cubic ZnMnO_3_ phase embedded in an amorphous ZnMnO_3_ matrix.

### Optical Properties of Composite Films

**Figure 5:**
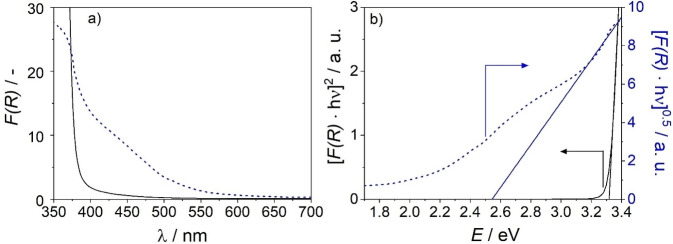
(a) UV/Vis diffuse reflectance spectra and (b) Tauc plots of a ZnO nanowire electrode (black lines) and of a ZnMnO_3_/ZnO nanocomposite film (blue lines). Linear regions in the Tauc plots are fitted and extrapolated to get an estimate of the band gap energy.

We have reported only recently that highly crystalline ZnMnO_3_ nanostructures featuring the defective cubic spinel phase and a two‐dimensional morphology grow on ZnO nanowires from identical precursor solutions upon reductive electrodeposition in the dark.^[31]^ For these crystalline ZnMnO_3_ nanosheets we estimated (upon supposition of an indirect semiconductor) a band gap energy of *E*_BG_=2.4±0.1 eV.^[31]^ In contrast to observations on these electrodeposited ZnMnO_3_ nanostructures,^[31]^ diffuse reflectance (DR) UV/Vis spectra of the photodeposits point to the presence of at least two types of electronic transition in the wavenumber range 400 nm<*λ*<550 nm (Figure 5a). The Tauc plot was derived from the UV/Vis diffuse reflectance spectrum of a ZnMnO_3_/ZnO composite film (photodeposition time: 45 min) and highlights a linear region between 3.1 and 3.4 eV (Figure 5b). The x‐intercept of a corresponding linear fit[^34^, ^35^] yields a band gap *E*_BG_ ~2.5 eV (*λ*~496 nm) in good accordance with the band gap of crystalline ZnMnO_3_ nanosheets. In addition, a second contribution at lower energies i. e. at *E*>2.1 eV can be observed. The lack of long range order in amorphous semiconductors gives rise to electronic states close to the conduction and valence band edges. We tentatively assign the low energy absorption to electronic transitions in the amorphous phase involving tail states.[^36^, ^37^] Thus, the observation of an (indirect) transition at *E*_BG_ ~2.5 eV together with light absorption down to energies of 2.1 eV affirm the presence of crystalline domains embedded in an amorphous matrix in the photodeposited ZnMnO_3_ phase.

### Capacitive Behavior of Composite Films

**Figure 6:**
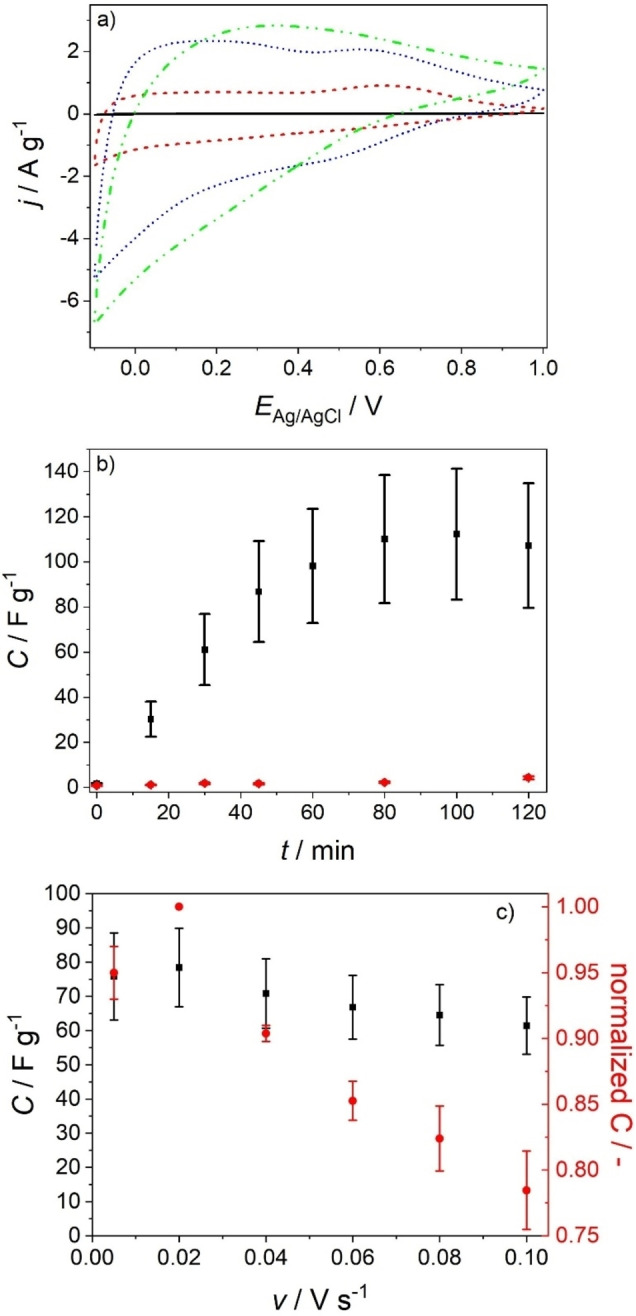
(a) Cyclic voltammograms of a nanowire electrode before (black, solid line) and after sequential photodeposition of ZnMnO_3_ (photodeposition potential: *E* ^OC^=0.376 V) for 15 min (red, dashed line), 45 min (blue, dotted line), and 120 min (green, dashed and dotted line); scan rate: *v*=0.020 V s^−1^. (b) Evolution of the specific capacitance (*C*) of the corresponding ZnMnO_3_/ZnO electrode (black) and of a ZnMnO_3_/ZnO composite electrode obtained by electrodeposition^[31]^ at *E*_Ag/AgCl_=0.376 V (red) upon stepwise increase of the deposition time. The specific capacitance was extracted from the second, stabilized voltammogram. (c) Specific capacitance (black squares) and normalized capacitance (red circles) as a function of scan rate for the ZnMnO_3_/ZnO electrode obtained by photodeposition for 45 min. The specific capacitance was extracted from cyclic voltammograms recorded with scan rates 0.005 V s^−1^≤*v*≤0.100 V s^−1^ (see Figure S3). For each scan rate, the second, stabilized voltammogram was taken for integration. The error bars were obtained by analyzing at least three different electrodes synthesized by following the same protocol. Electrolyte: 1 M Na_2_SO_4_ aqueous solution purged with N_2_.

The specific capacitance of ZnMnO_3_/ZnO electrodes in a 1 M Na_2_SO_4_ aqueous electrolyte was determined by cyclic voltammetry, galvanostatic and potentiostatic measurements. Capacitive currents give rise to approximately symmetric voltammograms at least for short photodeposition times (Figure 6a). The current density increases significantly (as compared to the bare ZnO nanowire electrode) upon photodeposition of ZnMnO_3_ (Figure 6a). Following photodeposition for *t*≤45 min, the cyclic voltammograms of ZnMnO_3_/ZnO electrodes feature two distinct broad peaks at *E*_Ag/AgCl_=0.20 V and 0.57 V (Figure 6a). We assign the peaks in the anodic scan to an increase of the oxidation state of Mn^3+^ and Mn^4+^ ions in the ZnMnO_3_ phase. The current density decreases at *E*_Ag/AgCl_≥0.6 V and drops to low current densities at *E*_Ag/AgCl_=1.0 V. Possible reasons for this behavior are (i) (almost) completely discharged ZnMnO_3_ deposits at *E*_Ag/AgCl_ ≥ 0.6 V, (ii) a high resistance of photodeposited ZnMnO_3_ at high positive potentials or (iii) slow ion extraction kinetics.

The capacitance (as extracted from cyclic voltammograms, see Supplementary Information) is as low as 1.2±0.5 F g^−1^ for ZnO nanowire arrays and increases linearly upon photodeposition up to a deposition time of 45 min (*C*=80±20 F g^−1^ at *v*=0.020 V s^−1^). The specific capacity was determined independently from the positive and negative going branch of the voltammogram yielding 100 C g^−1^ in both cases highlighting a Coulombic efficiency of 100 %. The high reversibility of the charging and discharging process points to the presence of a surface redox reaction. The capacitance levels off upon prolonged photodeposition and approaches a constant value for deposition times longer than 80 min (*C*=110±20 F g^−1^, Figure 6b). This saturation can be attributed to diffusion limitation. The accessibility of the porous structure provided by the ZnO nanowire array is essential for efficient electrolyte diffusion and charge compensation during electrochemical cycling. The reduction of the number and the volume of pores as observed by SEM for ZnMnO_3_/ZnO electrodes upon prolonged photodeposition (Figure 1d) will therefore give rise to a reduced accessibility of the porous structure for ions, thus slowing down charge compensation. To avoid any significant diffusion limitation, the photodeposition time was therefore limited to 45 min for all further experiments. Importantly, such ZnMnO_3_/ZnO electrodes feature a high rate capability maintaining at *v*=0.100 V s^−1^ 78±3 % of the highest capacitance (as determined at *v*=0.020 V s^−1^, Figure 6c).

**Figure 7:**
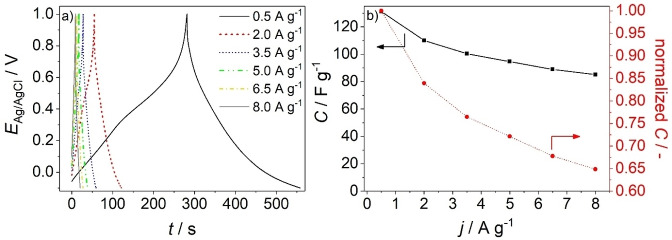
(a) Galvanostatic charging‐discharging curves of a ZnMnO_3_/ZnO electrode (photodeposition time: 45 min) recorded at different current densities. (b) Specific capacitance and normalized capacitance of the corresponding electrode. Electrolyte: 1 M Na_2_SO_4_ aqueous solution purged with N_2_.

Galvanostatic measurements were performed to investigate in more detail the charging and discharging behavior of ZnMnO_3_/ZnO electrodes (Figure 7a). The curves show an approximately linear behavior. Deviations can be associated with faradaic reactions characteristic of pseudocapacitive materials.[^38^, ^39^, ^40^]

The capacitance as estimated from galvanostatic measurements (see Supplementary Information) accounts for 130±20 F g^−1^ at *j*=0.5 A g^−1^ (Figure 7b) corresponding to a discharge capacity of 140 C g^−1^. A capacitance preservation of ~65 % at a current density *j*=8.0 A g^−1^ (as compared to the capacitance determined at *j*=0.5 A g^−1^) is observed (Figure 7b) and highlights again the high rate capability of ZnMnO_3_/ZnO electrodes. This high rate capability (as observed both in voltammetric and in galvanostatic experiments, Figures 6c and 7b, respectively) is connected to the high porosity of the ZnMnO_3_ shell featuring nanocrystalline domains embedded in an amorphous ZnMnO_3_ matrix (Figures 3 and S2) thus providing a high specific surface area and short diffusion paths of ions in the ZnMnO_3_ electroactive phase. Furthermore, only a minor voltage drop (*iR*‐drop) is observed in the galvanostatic charging – discharging curves when switching from charging to discharging (Figure 7a). It is well‐known that crystalline matter features an enhanced electronic conductivity compared to amorphous matter.[^39^, ^41^] The slow‐down of electron transport in amorphous matter results from a low electron mobility as charge carriers are scattered at local defects and are trapped multiple times in localized states, which form the so called band tails in amorphous semiconductors.^[41]^ Importantly, however, the ZnMnO_3_ shell, which is conformally coating the ZnO nanowires, is very thin (~20 nm, Figure 3). The short electron diffusion distance may thus compensate for the lower electronic conductivity within the amorphous matrix. In addition, ZnO nanowires have been shown to feature high carrier densities.^[32]^ This together with the specific nanowire morphology assures a high electronic conductivity. The small *iR*‐drop may thus be attributed to the specific morphology of the ZnMnO_3_ shell and of the overall composite film, respectively.

More details about the kinetics of charge storage in the amorphous ZnMnO_3_ matrix containing nanocrystalline domains is obtained by recording current transients upon charge accumulation and extraction (Figure S4). Cyclic voltammetry relies on a relatively fast variation of potential with time (in this study: *v*=0.005–0.100 V s^−1^). Accordingly, currents depend on both scan rate and applied potential in cyclic voltammetry. Due to this convolution, it is complex to extract slow kinetics from this type of measurements.^[42]^ In potentiostatic measurements, by contrast, even processes featuring slow kinetics can be observed at a specific potential as the potential value is kept constant for a defined time^[42]^ (here *t*=150 s).

The specific capacitance of ZnMnO_3_/ZnO electrodes obtained by photodeposition for 45 min (as estimated from potentiostatic measurements, see Supplementary Information) is 160±40 F g^−1^ (corresponding to a discharge capacity of 200 C g^−1^) and hence almost twice as high as the capacitance extracted from cyclovoltammetric measurements (*C*=80±20 F g^−1^ at *v*=0.020 V s^−1^). Clearly, potentiodynamic measurements do not sample the whole electroactive fraction of the ZnMnO_3_ phase. The higher specific capacitance extracted from potentiostatic cycling points to a second charge storage mechanism (in addition to a fast surface redox reaction), which is associated with a much slower kinetics and which possibly involves subsurface regions of the electroactive phase. In line with this interpretation, the discharge curve (Figure S4b) points to the contribution of at least two processes with different kinetics.

### Photo‐versus Bias‐induced Generation of Reactive Charge Carriers

**Figure 8:**
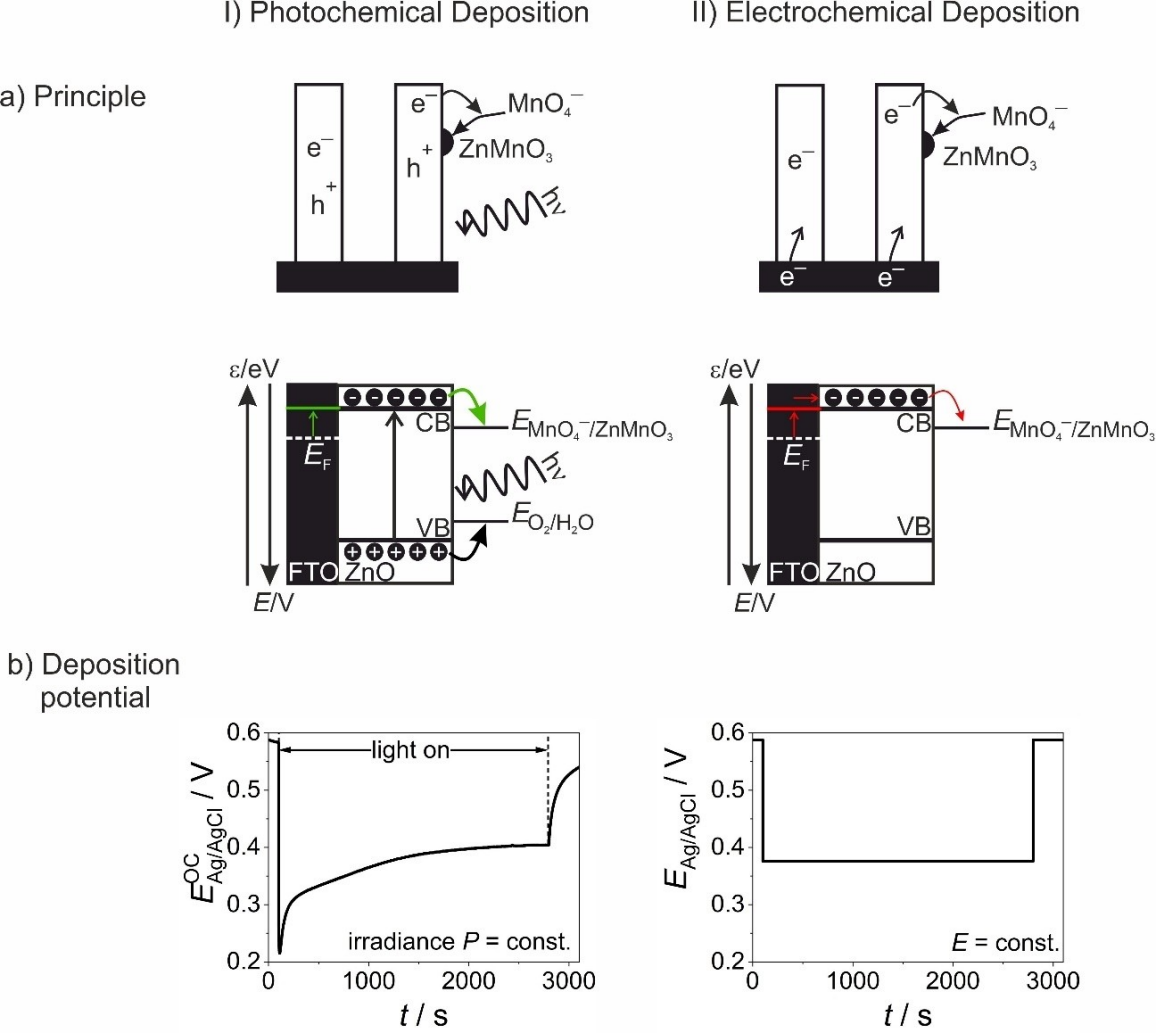
(a) Schemes of a ZnO nanowire electrode and corresponding band schemes highlighting the principle of the light‐induced generation of electrons and holes (a(I)) or the bias‐induced injection of electrons (a(II)) in the semiconductor as well as the consecutive interfacial charge transfer reactions, respectively (CB…conduction band, VB…valence band). (b) The deposition potential is tracked upon photodeposition (at a constant light irradiance *P* and at open circuit conditions, b(I)) and it is externally set to a constant value upon electrodeposition (b(II)), respectively. The photodeposition potential (i. e. the open circuit potential E^OC^, b(I)) has been measured upon UV/Vis exposure of a ZnO nanowire electrode in aqueous 0.175 mM KMnO_4_ solution. Irradiance: *P*=330 mW cm^−2^.

The photoinduced deposition of ZnMnO_3_ results from charge transfer reactions at the semiconductor/electrolyte interface involving photogenerated electrons and, possibly, photogenerated holes (Figure 8a(I)). Only recently, we reported on the reductive electrodeposition (i. e. bias‐induced deposition in the absence of UV/Vis light) of highly crystalline ZnMnO_3_ nanosheets from an identical aqueous KMnO_4_ solution onto ZnO nanowire arrays at room temperature.^[31]^ In this case, reactive electrons were injected into ZnO upon the application of a sufficiently negative electrode potential followed by an interfacial electron transfer to permanganate being a strong oxidizing agent. This bias‐induced ZnMnO_3_ deposition (Figure 8a(II)) was attributed to the electrochemical reduction of permanganate at the surface of the reactive ZnO substrate, which acts at the same time as the Zn precursor
(1)

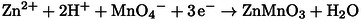




It remained unclear, however, whether the Zn^2+^ ions necessary for the reaction are provided by ZnO dissolution or whether ion diffusion from the buried ZnMnO_3_/ZnO interface to the reactive ZnMnO_3_/electrolyte interface takes place. Irrespective of this, a surface‐enabled epitaxial process was identified as the fundamental reason for the observed crystallization of ZnMnO_3_ at room temperature.^[31]^

In the case of photoinduced deposition, the fate of photogenerated holes has to be included in the description of the reactive process. Photogenerated holes in ZnO are able to oxidize water at the semiconductor/electrolyte interface (Figure 8a(I)). In addition, photocorrosion of ZnO[^43^, ^44^]

(2) $$ZnO\;+\;y\;H_{2}O\;+\;2\;h^{+}\to {\left[Zn{\left(OH\right)}_{y}\right]}^{(2-y)+}\;+\;0.5\;O_{2}\;+\;y\;H^{+}$$

has to be taken into account. On the one hand, this process will therefore contribute to the local concentration of zinc precursor species at the reactive semiconductor/electrolyte interface. On the other hand, photocorrosion will lead to surface roughening at an atomic level, which will impede epitaxial growth of extended nanostructures as previously observed in the case of bias‐induced deposition.^[31]^ High local concentrations of zinc precursor species, photogenerated electrons and permanganate will give rise to a high ZnMnO_3_ formation rate. This together with the absence of atomically flat surfaces on photocorroding ZnO nanowires may therefore explain the formation of the unique ZnMnO_3_ structure consisting of crystalline nanodomains embedded in a porous and amorphous matrix.

The photodepostion process can be tracked *in situ* by open circuit potential measurements (Figure 8b(I)). As ZnO is an n‐type semiconductor,^[45]^ photogenerated holes can be transferred – at least partially – to water.^[46]^ Given that the interfacial transfer is faster for holes than for electrons, a displacement of the Fermi level, *E*_F_, towards the conduction band will take place as a consequence of electron accumulation in the semiconductor. For crystallites sustaining band bending this displacement will occur at the interfacial depletion layer.^[47]^ The extent of band bending inside the ZnO nanowires is thus reduced upon light exposure due to the increase of the electron concentration. Consequently, the probability for electron transfer to acceptor species at the semiconductor/electrolyte interface increases. In addition, electrons photogenerated near the semiconductor/electrolyte interface can directly be transferred to solution species particularly in the presence of efficient electron acceptors (such as MnO_4_^−^).

If the semiconductor film is in electrical contact with a conducting substrate, the photoinduced quasi‐Fermi level shift can be measured as a change of the open circuit potential. Accordingly, the chronopotentiometric profile measured upon photodeposition (Figure 8b(I)) features a pronounced decrease upon light exposure highlighting electron accumulation in the semiconductor. Following an initial spike, the open circuit potential slightly increases again. This increase can be rationalized by a lowering of the hole transfer rate – and a concomitant enhancement of electron/hole recombination – once the surface is being covered by the photodeposit. Finally, the open circuit potential reaches a constant value (Figure 8b(I)). This steady state is governed by the kinetic competition between electron/hole pair generation and recombination as well as electron transfer to MnO_4_^−^, respectively. We identify the corresponding stationary open circuit potential with the photodeposition potential in this study.

Interestingly, deposit formation is very slow if the electrode potential of a ZnO nanowire array is set (in an identical electrolyte solution) to the value of the photodeposition potential (i. e. to *E*_Ag/AgCl_=0.376 V) by the application of an external bias in the absence of UV light (a process corresponding to electrodeposition).^[31]^ A significant increase of the capacitive current density (as compared to the pristine ZnO nanowire array) after 120 min of electrodeposition has previously been evidenced by cyclic voltammetry.^[31]^ However, the specific capacitance of the resulting ZnMnO_3_/ZnO composite electrodes is much lower than for composite electrodes obtained by photodeposition at the same deposition potential (Figure 6b). Specifically, there is only a small increase of the specific capacitance upon electrodeposition at *E*_Ag/AgCl_=0.376 V from 1.2±0.5 F g^−1^ (for pristine ZnO nanowire arrays) to 4.2±0.7 F g^−1^ (after an electrodeposition time of 120 min), whereas a huge increase is observed for ZnMnO_3_/ZnO electrodes photodeposited at the same potential (107±28 F g^−1^ for *t*=120 min). It has to be mentioned that a constant potential is applied during electrodeposition (Figure 8b(II)), whereas the photodeposition potential varies in the initial deposition stage (while light irradiance is kept constant) until reaching a constant value (Figure 8b(I)). However, the specific capacitance of ZnMnO_3_/ZnO electrodes synthesized by photodeposition is ~25 times higher than the specific capacitance of electrodeposited electrodes (photo‐ and electrodeposition times: *t*=120 min). In line with this observation and as reported previously,^[31]^ only a very low amount of ZnMnO_3_ is electrodeposited at *E*_Ag/AgCl_=0.376 V. While mechanistic differences between photo‐ and electrodeposition have to be considered, it also gets clear that electrode potential is not a good descriptor for the concentration of reactive electrons during photodeposition i. e. under dynamic conditions, where both photogenerated holes and electrons contribute to the reactive event. Obviously, there is a high probability for electrons photogenerated near the semiconductor/electrolyte interface to transfer to the strong oxidizing agent MnO_4_^−^. These electrons will thus not contribute to the modification of the quasi‐Fermi level. Indeed, much more negative potentials are required to electrodeposit significant amounts of ZnMnO_3_.^[31]^ However, even after optimized electrodeposition at *E*_Ag/AgCl_=0.000 V^[31]^ only a low capacitance of *C*≤20 F g^−1^ has been obtained. This can be rationalized by the very different morphology and crystallinity of the ZnMnO_3_ phase resulting from the two different deposition approaches. Indeed, highly crystalline ZnMnO_3_ nanosheets (featuring lateral dimensions of up to 100 nm and a thickness<10 nm) are electrodeposited at the ZnO/water interface as a result of an epitaxial growth process. ^[31]^ While the electronic conductivity of the electroactive phase will be enhanced due to the high crystallinity, its beneficial effect on electron conduction may at least partially be thwarted by the larger electron diffusion path resulting from the high lateral extension of the nanosheets. More importantly, lower ion diffusion coefficients in the crystalline structure (as compared to the amorphous phase)[^48^, ^49^] will be detrimental to the specific capacitance of the composite electrode if subsurface regions also contribute to overall charge storage. In this context, it has been reported that amorphous or mixed crystalline and amorphous electroactive phases feature important advantages.[^6^, ^30^, ^50^, ^51^] Due to the structural disorder amorphous phases typically provide more percolation pathways for efficient ion diffusion than crystalline phases. In addition, they may provide an increased number of active sites for pseudocapacitive reactions. The optimization of both ion and electron transport in a pseudocapacitive material can be realized by designing crystalline‐amorphous heterostructures. Furthermore, additional charge storage sites at the interface between crystalline and amorphous domains may give rise to an increased specific capacitance.[^5^, ^52^] The slow process contributing to the charging/discharging of the crystalline–amorphous biphasic ZnMnO_3_ photodeposit may indeed point to the involvement of related interfacial sites. However, more investigations would be necessary to clarify the underlying principle.

### Electrodeposition at Different pH Values – Impact on Morphology and Charge Storage Behavior of ZnMnO_3_/ZnO Composite Electrodes

Elucidation of the mechanistic details of ZnMnO_3_ photodeposition on ZnO nanowires is complicated by the fact that reductive processes (see e. g. Equation 1; electron transfer to residual oxygen…) and oxidative processes (see e. g. Equation 2; photocorrosion of ZnO…) together with additional reactions (such as the dissolution of ZnO) occur in parallel on the substrate surface. The complexity of the deposition process is reduced upon electrodeposition due to the spatial separation of reductive processes (taking place at the reactive ZnO/electrolyte interface) from oxidative processes (taking place at the counter electrode) in the electrochemical cell. One critical process parameter for both photo‐ and electrodeposition is the local pH value near the reactive interface, which may change dynamically due to proton generation/consumption in different reaction steps (see e. g. Equations 1 and 2).

To evaluate the importance of proton concentration for deposit formation, we used precursor solutions featuring different pH values (pH 4, pH 7 and pH 10). Taking into consideration that the variation of one process parameter may impact many different reaction steps, we opted for reducing the complexity of the reactive system by performing electrodeposition. This approach aims at identifying mechanistic reasons for the observed relationship between processing conditions and the physicochemical and functional properties of the resulting composite electrodes.

**Figure 9:**
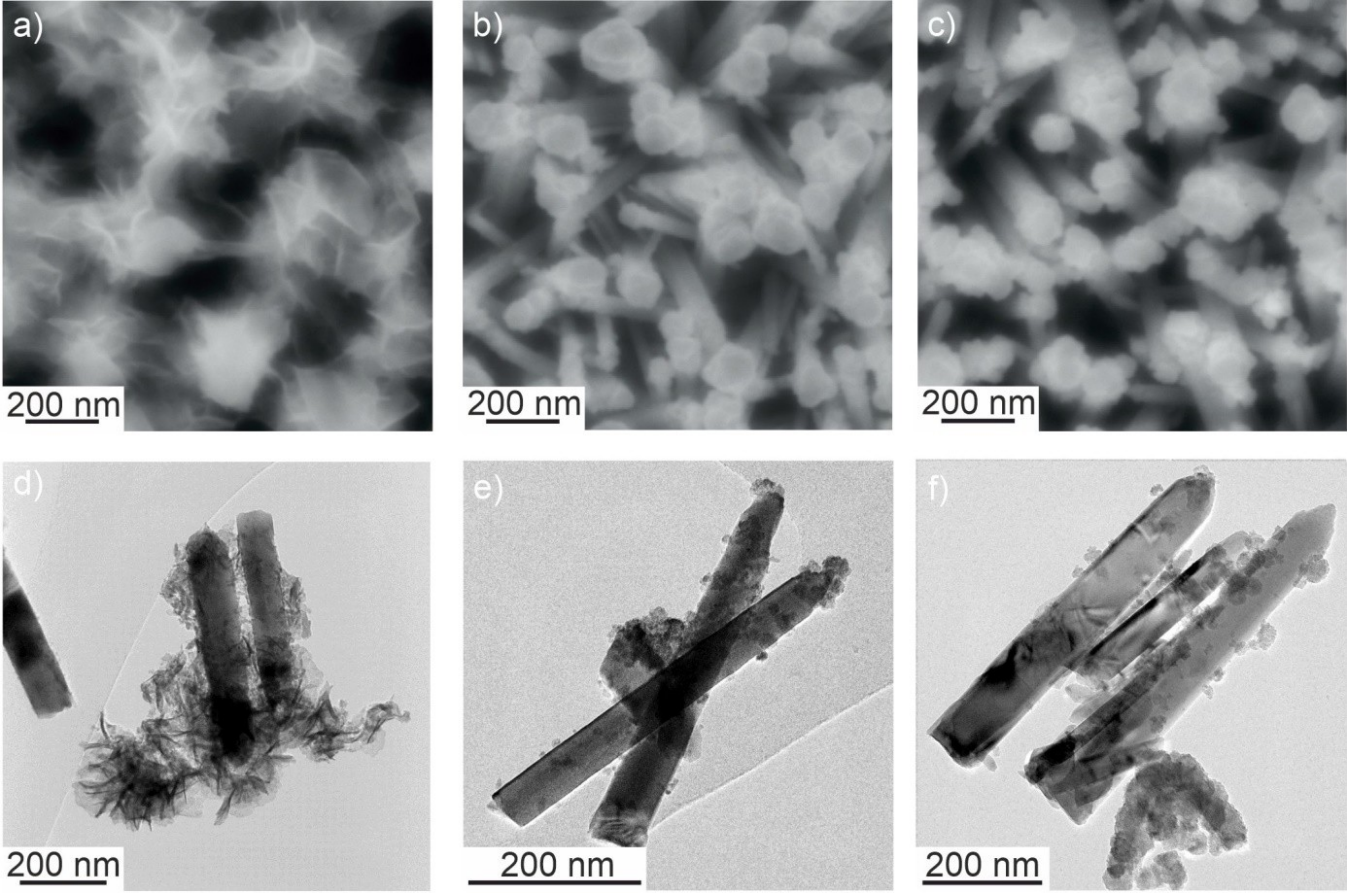
Top view scanning electron micrographs (a–c) and transmission electron micrographs (d–f) of ZnMnO_3_/ZnO composite electrodes after 45 min of electrodeposition at pH 4 (a,d), pH 7 (b,e) and pH 10 (c,f). Sample preparation for TEM analysis (see Supporting Information) implicates mechanical disruption of the porous film, which may lead to the detachment of the ZnMnO_3_ deposit from the ZnO nanowires (see (d) and (f)).

Top view (Figure 9a–c) as well as cross section (Figure S5) scanning electron micrographs of composite films reveal the presence of an electrodeposited phase mainly in nanowire top regions. The morphology of the deposits strongly depends on the pH value of the precursor solution as corroborated by transmission electron microscopy (Figure 9d–f). Whereas a sheet‐like morphology is obtained for a precursor solution at pH 4 (Figure 9a,d), particle‐like deposits are observed for composite films resulting from electrodeposition at pH 7 (Figure 9b,e) and pH 10 (Figure 9c,f), respectively.

High‐resolution TEM images (Figure S6b,e,h) evidence that the newly formed phase is crystalline as lattice fringes are discernible for all deposits and are clearly visible in the FFT patterns (Figure S6c,f,i). The corresponding lattice spacings (Table S2) are in good agreement with the defective cubic spinel structure of ZnMnO_3_.^[28]^ The elemental composition of the electrodeposits was investigated by EDX analysis. The decoration of the top regions of ZnO nanowires is clearly visible in STEM‐HAADF images (Figure S7a,e,i). Both zinc and manganese are contained in the newly formed phase as visible from the elemental map of the composites (Figure S7). The quantification of Mn and Zn present in the electrodeposited phase yields an approximate Mn to Zn atomic ratio of 1 : 1 independent from the pH value of the precursor solution. This elemental ratio together with the lattice spacings deduced from FFT patterns (Table S2) evidence the electrodeposition of the defective cubic ZnMnO_3_ phase onto the surface of ZnO nanowires at room temperature.

Proton concentration may influence electrodeposit formation in different ways. On the one hand, the electronic energetics of the semiconductor oxide i. e. the absolute valence and conduction band positions vary systematically with solution pH due to surface protonation and deprotonation reactions or as a consequence of proton uptake in subsurface regions.^[53]^ In this context, adjustment of the electrodeposition potential by ±0.059 V per pH unit aims at keeping the relative position of the Fermi level in the semiconductor constant with respect to the conduction band edge (see Experimental section). However, a pH‐induced change of the surface charge may significantly influence the interaction of charged precursor species in solution (e. g. MnO_4_^−^ ions) with the surface thus impacting the interfacial reaction. In addition, the limited chemical stability of ZnO leading to its dissolution in acidic and alkaline media has to be taken into account. Partial dissolution of ZnO will lead to the evolution of dynamically changing surface structures and possibly to surface roughening. Epitaxial growth of extended ZnMnO_3_ nanosheets at the lateral surfaces of ZnO nanowires, however, requires atomically flat surfaces as highlighted in our previous study.^[31]^ On the other hand, ZnO dissolution will lead to an increased concentration of ionic zinc species at the reactive interface, which most probably act as precursor for ZnMnO_3_ deposition.

**Figure 10:**
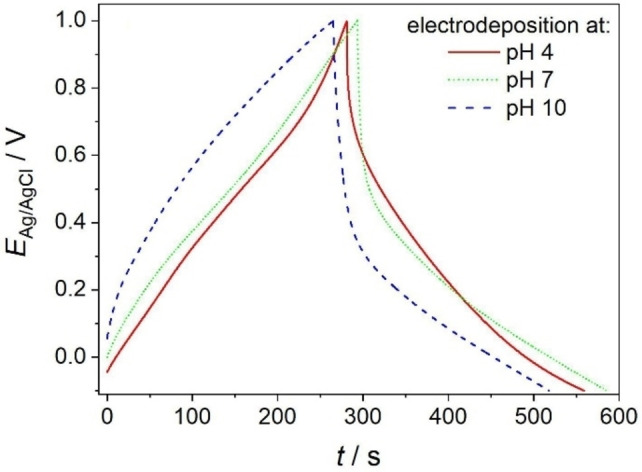
(a) Galvanostatic charging‐discharging curves recorded at low current densities for ZnMnO_3_/ZnO composite electrodes electrodeposited at pH 4 (*j*=0.150 A g^−1^), pH 7 (*j*=0.125 A g^−1^) and pH 10 (*j*=0.100 A g^−1^). Electrolyte: 1 M Na_2_SO_4_ aqueous solution purged with N_2_.

Galvanostatic measurements (Figures 10 and S8) as well as cyclic voltammetry (Figure S9) were performed to investigate the charging and discharging behavior of electrodeposited ZnMnO_3_/ZnO electrodes. For composite films resulting from electrodeposition at pH 4, galvanostatic charging‐discharging curves show an approximately linear behavior characteristic of pseudocapacitive materials.[^3^, ^54^] A significant deviation from linearity is observed for films electrodeposited at pH 7 and pH 10. Furthermore, these films show significant IR drops when switching from charging to discharging in contrast to films resulting from electrodeposition at pH 4, which feature only a minor voltage drop (Figure 10). The IR drop results from Ohmic resistances in the system such as the ionic resistance in the electrolyte, the electronic resistance inside the material, and interfacial resistances. Electrodeposits grown at the surface of ZnO nanowires at varying pH values have the same crystal structure and composition but feature significant morphological differences. We therefore attribute the pronounced IR drop observed for films deposited at pH 7 and pH 10 to a low electronic conductivity within aggregates of crystalline ZnMnO_3_ nanoparticles and/or to a high interfacial resistance between ZnMnO_3_ nanoparticle aggregates and ZnO nanowires. For films resulting from electrodeposition at pH 5^[31]^ and pH 4 (Figures 9a,d and S6b), however, the electrodeposit consists of crystalline nanosheets with lateral dimensions of up to 100 nm, which are connected to the ZnO nanowires. This morphology results from epitaxial growth of ZnMnO_3_ on the lateral facets of ZnO nanowires^[31]^ and assures effective electronic communication between the electroactive material and the conducting scaffold. Furthermore, extended crystalline nanosheets allow for an electron transport in the electroactive ZnMnO_3_ phase, which is not slowed down by grain boundary effects^[55]^ as in the case of strongly aggregated ZnMnO_3_ nanoparticle deposits (resulting from electrodeposition at pH 7 and pH 10). This together with a low interfacial resistance between the electroactive ZnMnO_3_ and the ZnO substrate gives thus rise to the observed minor voltage drop in galvanostatic curves of the ZnMnO_3_ nanosheet/ZnO nanowire composite electrode. In addition, the nanowire morphology assures a high interfacial area between the electroactive material and the electrolyte, which is beneficial for interfacial charge storage and possibly facilitates cation insertion into subsurface regions of the electroactive material.

In line with observations from galvanostatic measurements, films electrodeposited at pH 4 are characterized by virtually symmetric cyclic voltammograms (Figure S9a). Two distinct broad peaks at *E*_Ag/AgCl_ ~0.20 V and *E*_Ag/AgCl_ ~0.65 V are present in the anodic scan. On the contrary, a highly resistive behavior is manifested by the cyclic voltammograms of films deposited at pH 7 and pH 10.^[56]^ These electrodes are therefore excluded from the further discussion.^[54]^

In line with our previous study on ZnMnO_3_ nanosheet/ZnO nanowire films (resulting from electrodeposition in pure 0.175 mM KMnO_4_ aqueous solution at pH 5),^[31]^ we observe only a very modest charge storage behavior for the composite electrode resulting from electrodeposition at pH 4 (Figure S10). In 1 M Na_2_SO_4_ aqueous solution, a specific capacitance (as referenced to the total electrode mass i. e. the mass of ZnO nanowires and of the ZnMnO_3_ deposit) of *C*=36 F g^−1^ was estimated from the galvanostatic measurement at *j*=0.15 A g^−1^. This is much lower than the capacitance of a composite film resulting from photodeposition and consisting of ZnO nanowires covered by a crystalline–amorphous biphasic ZnMnO_3_ shell (*C*=130 F g^−1^ at *j*=0.50 A g^−1^). In addition, a much lower rate capability is observed for the ZnMnO_3_ nanosheet/ZnO nanowire electrodes with a capacitance preservation of ~45 % at a current density *j*=0.40 A g^−1^ (as compared to the capacitance determined at *j*=0.15 A g^−1^, Figure S10b). These observations add importance to the unique biphasic nanostructure accessible via photodeposition. While the reduced complexity of electrodeposition may help to elucidate some mechanistic details of deposit formation at the reactive interface between the ZnO substrate and the aqueous KMnO_4_ solution, such highly complex nanostructures can not be achieved without the mechanistic complexity inherent to the photodeposition process.

### General Discussion

The ZnMnO_3_/ZnO composite films prepared by photodeposition do not feature any record‐breaking capacitance, however, the performance is comparable to some previously reported nanostructures. As an example, Yan et al.^[57]^ prepared composite films consisting of crystalline SnO_2_ nanowires, which were coated by an amorphous MnO_2_ shell. The specific capacitance (as based on the mass of MnO_2_) of the resulting electrodes in 1 M Na_2_SO_4_ aqueous solution accounted for 637 F g^−1^ (as extracted from cyclic voltammograms recorded at *v*=2 mV s^−1^) and for 800 F g^−1^ (as determined by galvanostatic cycling at *j*=1.0 A g^−1^). These values convert to 108 F g^−1^ and 136 F g^−1^ when referred to the mass of the whole porous film i. e. the electroactive phase (MnO_2_) and the porous substrate (i. e. the SnO_2_ nanowires), respectively.

Given the enormous potential of crystalline–amorphous biphasic nanostructures in charge storage applications, an urgent need for synthesis routes towards these materials has been identified only recently.^[30]^ Such synthesis routes shall allow not only for an optimization of the materials’ functional properties, but may furthermore help to better understand the physicochemical principles underlying the improved performance. The photodeposition approach towards ternary metal oxide nanostructures presented in this study is a step in this direction.

It remains to be elucidated, whether the successful photodeposition process is unique to the investigated material combination or may be extended to other substrate/deposit systems thus providing a basis for a more general synthesis approach yielding crystalline–amorphous biphasic oxide nanostructures. In any case, we are convinced that the photodeposition approach presented here (together with the electrodeposition approach reported here as well as in a recent study)^[31]^ constitutes a very promising green synthesis strategy for the rational design of high performance electroactive materials.

## Conclusions

Conformal ZnMnO_3_ shells were photodeposited on ZnO nanowires from aqueous KMnO_4_ solution at room temperature. The morphology, the crystallinity and crystal structure, the optical properties and the capacitive behavior of the resulting composite electrodes have been characterized and the following conclusions have been reached:

- The photodeposited phase consists of a porous and amorphous ZnMnO_3_ matrix embedding nanocrystalline domains, which feature the defective cubic spinel ZnMnO_3_ structure.
- The photodeposition time governs the thickness of the ZnMnO_3_ shell and thus the accessibility of the porous structure provided by the ZnO nanowire array for ions from solution. A photodeposition time of 45 min yields (under the investigated experimental conditions) a composite film assuring efficient ion diffusion.
- Corresponding composite electrodes feature a specific capacitance, which depends strongly on the electrochemical method which is used for capacitance determination yielding *C*=80±20 F g^−1^ (as extracted from cyclic voltammograms measured at *v*=0.020 V s^−1^), *C*=130±20 F g^−1^ (as estimated from galvanostatic cycling at *j*=2.0 A g^−1^) and *C*=160±40 F g^−1^ (as estimated from potentiostatic measurements) in 1 M Na_2_SO_4_ aqueous solution.
- The mechanism of photodeposition differs significantly from the mechanism of electrodeposition at the same deposition potential (and using identical precursor solutions and ZnO nanowire arrays as substrates). The mechanistic differences result from differences in charge injection/generation and interfacial transfer and give rise to composite films featuring very different physicochemical properties.
- The complexity of the deposition process can be reduced by performing electrodeposition due to the spatial separation of reductive and oxidative processes, which may help to elucidate some mechanistic details of deposit formation.
- The results substantiate the high potential of photodeposition as an alternative and green route towards functional materials.

## Supporting Information Summary

Further experimental details, scheme of the three–electrode photo(electro)chemical cell, additional high resolution transmission electron micrographs and FFT patterns of ZnMnO_3_/ZnO composites, cyclic voltammograms recorded at different scan rates, current transients recorded upon electron accumulation and extraction, scanning electron and transmission electron micrographs as well as elemental intensity maps of ZnMnO_3_/ZnO composites resulting from electrodeposition, galvanostatic charging‐discharging curves of composite electrodes resulting from electrodeposition, a comparison of experimentally determined lattice spacings of the photodeposited and electrodeposited phases with literature values. An additional reference^[58]^ is cited within the Supporting Information.

## Conflict of interest

The authors declare no conflict of interest.

1

## Supporting information

As a service to our authors and readers, this journal provides supporting information supplied by the authors. Such materials are peer reviewed and may be re‐organized for online delivery, but are not copy‐edited or typeset. Technical support issues arising from supporting information (other than missing files) should be addressed to the authors.

Supporting Information

## Data Availability

The data that support the findings of this study are available from the corresponding author upon reasonable request.
