# Supplementary material for: Conformal Coverage of ZnO Nanowire Arrays by ZnMnO3: Room‐temperature Photodeposition from Aqueous Solution
Source: Chemphyschem. 2023 Sep 22;24(21):e202300250. doi: 10.1002/cphc.202300250 (PMC10962551; doi:10.1002/cphc.202300250)
Supplement: Supplementary file 1 — Supporting Information [file CPHC-24-0-s001.pdf]

ChemPhysChem

Supporting Information

Conformal Coverage of ZnO Nanowire Arrays by $ZnMnO_3$: Room-temperature Photodeposition from Aqueous Solution

Karin Rettenmaier, Gregor A. Zickler, and Thomas Berger*

Further Experimental Details

Electrodeposition of ZnO Nanowires

FTO (fluorine-doped tin oxide, SnO_2:F)–coated glass substrates (Pilkington TEC 8, resistance 8 Ω/□) were used as conducting substrates for the electrodes. A dense AZO (aluminum-doped zinc oxide, ZnO:Al)–layer was sputtered onto the FTO–substrates to facilitate the adhesion of ZnO nanowires grown by electrochemical deposition and guarantee a homogeneous ZnO nanowire coverage.[1–3] For ZnO nanowire electrodeposition, a three–electrode cell consisting of the AZO/FTO–coated glass substrate as working electrode, a flat Pt spiral as counter electrode and an Ag/AgCl (1 M KCl) reference electrode (PalmSens) was used. The AZO/FTO–substrate was covered with Teflon tape to define the area (2.25 cm^2) exposed to the electrolyte. ZnO nanowire deposition was performed at T = 80 °C in an oxygen (O_2 5.0)–purged 1 M KCl (Sigma Aldrich, purity ≥ 99.0 %) and 0.5 mM $ZnCl_2$ (Sigma Aldrich, anhydrous, purity ≥ 98 %) aqueous solution (V = 135 mL). An electrodeposition potential E = -1.026 V vs. Ag/AgCl (1 M KCl) reference electrode was applied until a total charge of 14.0 C cm^2 had passed. The resulting ZnO nanowire array was thoroughly rinsed with ultrapure water and dried at room temperature in air.

Sample Preparation for Transmission Electron Microscopic (TEM) Analysis

Sample preparation for TEM analysis consisted of scratching one part (around one third) of the $ZnMnO_3$/ZnO composite film off the substrate with a razor blade. The obtained powder was dispersed in absolute ethanol (800 µL, Merck, 99.95 %) and ultrasonicated in a water bath at room temperature for 15 min. Subsequently, 7 µL of the dispersion were transferred to the topside of an Au-supported lacey carbon grid placed on top of a standard laboratory filter paper. This procedure was repeated twice. Prior to TEM measurements and after establishing a base pressure p_{base} = 0.08 mbar, the Au lacey carbon grids loaded with the specimen were treated for 45 s in a He plasma (p_{He} = 0.3 mbar, power: P = 20 W, f = 40 kHz; Zepto CE 40 kHz, Diener electronic – Plasma-Surface-Technology).

Evaluation of the Capacitive Behavior

Capacitance Determination from Cyclic Voltammetry

The specific capacitance C (in F g^{-1}) was extracted from cyclic voltammograms as recorded with a sweep rate v in the potential range $E_0 \leq E \leq E_1$ (and $E_1 - E_0 \equiv \Delta E$) by using Equation S1 [4]

$$C = \frac{1}{2 \cdot v \cdot \Delta E \cdot m} \int_{E_0}^{E_1} (i_a + |i_c|) dE \qquad \text{(Equation S1)}$$

where i_a and i_c is the current of the anodic and cathodic branch of the cyclic voltammogram, respectively, and m is the electrode mass. The electrode mass corresponds to the sum of the masses of the photodeposited, electroactive phase (i.e. $ZnMnO_3$) and of the porous substrate (i.e. the ZnO nanowire array), respectively. Correspondingly, the specific capacity (in C g^{-1}) was calculated by integration of the positive- or negative-going branch of the cyclic voltammogram.

Capacitance Determination from Galvanostatic Measurements

Galvanostatic charging and discharging was used to estimate the capacitance, C (in F g^{-1}). The charge passed during the anodic or cathodic scan ($i \cdot \Delta t$) was divided by the mass, m, of the composite film and by the potential window ΔE of the galvanostatic cycle (Equation S2)

$$C = \frac{i \cdot \Delta t}{\Delta E \cdot m} \qquad \text{(Equation S2)}$$

where i is the current and Δt is the time of charging or discharging, respectively. The specific capacity (in C g^{-1}) was calculated by referring the charge to the mass of the composite film.

Capacitance Determination from Potentiostatic Experiments

First, current transients were recorded upon stepping the electrode potential from $E_{Ag/AgCl}$ = -0.1 V to 1.0 V and back. The specific capacitance (in F g^{-1}) was then obtained by integrating the current transient corresponding to discharging over time (t = 150 s) and referring the calculated charge to the potential window ΔE = 1.1 V and the mass, m, of the composite film

$$C = \frac{\int i \cdot dt}{\Delta E \cdot m} \qquad \text{(Equation S3)}$$

The specific capacity (in C g^{-1}) was calculated by referring the charge to the mass of the composite film.

Supplementary data

Additional Figures

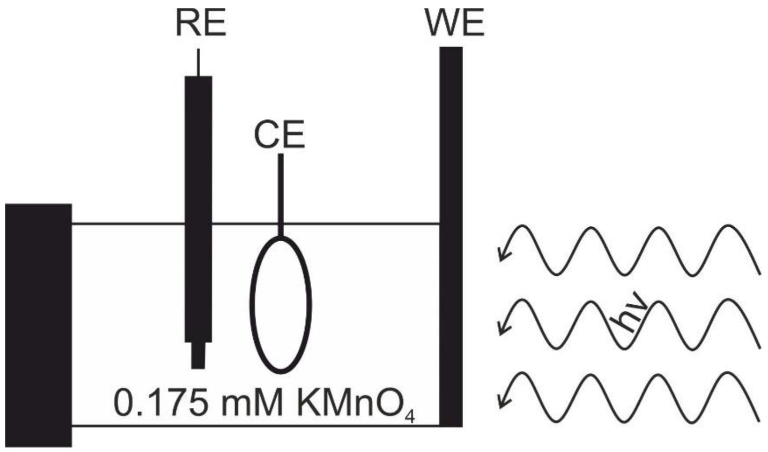

Figure S1: Scheme of the three–electrode photo(electro)chemical cell used for the deposition of $ZnMnO_3$ on ZnO nanowire arrays. WE…working electrode (ZnO nanowire array), RE…reference electrode (Ag/AgCl (1 M KCl) electrode). For electrodeposition a Pt counter electrode (CE) was used.

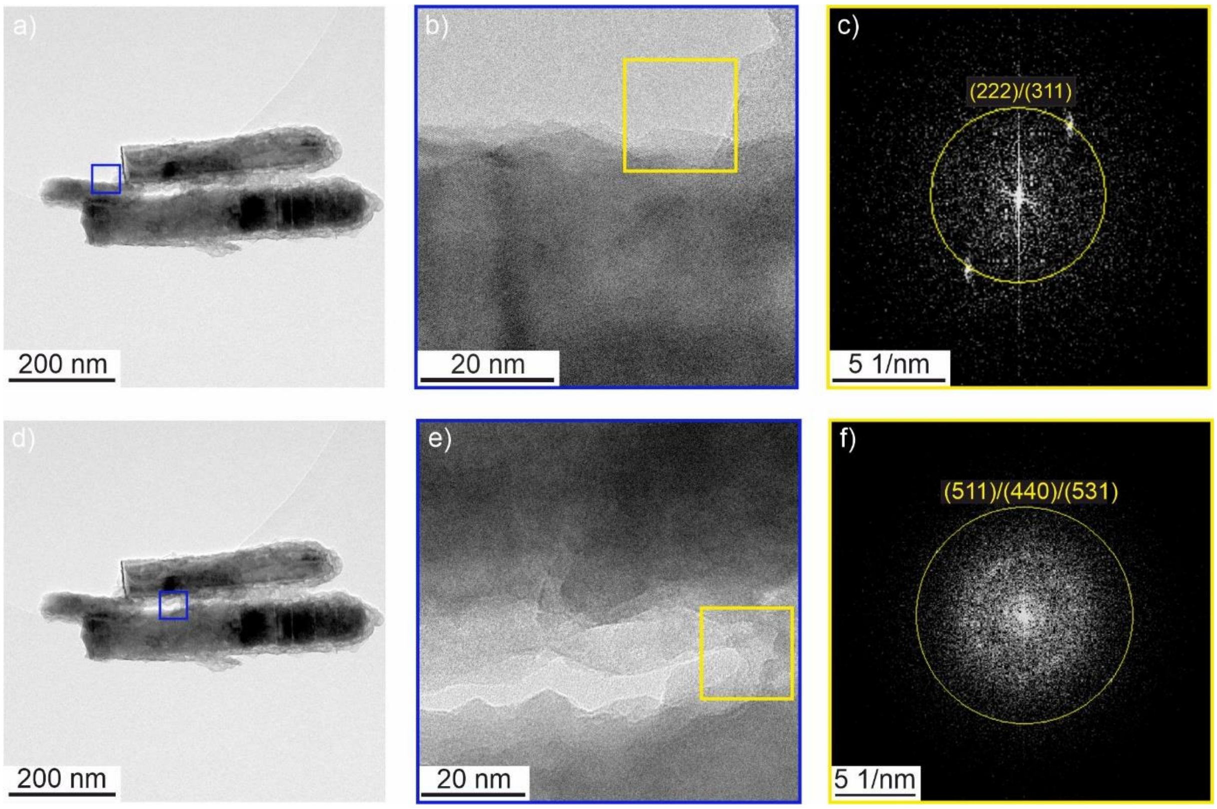

Figure S2: (a,d) Transmission electron micrographs, (b,e) High resolution transmission electron micrographs and (c,f) FFT patterns of $ZnMnO_3$/ZnO composites obtained by photodeposition (photodeposition time: *t* = 45 min). The blue squares in (a) and (d) indicate the position, at which the HRTEM image of the $ZnMnO_3$ phase (b and e) is taken. FFT analysis of the $ZnMnO_3$ phase (c and f) is performed on the area marked by a yellow square in the HRTEM images (b and e). Indexing is based on the analysis of the FFT pattern.

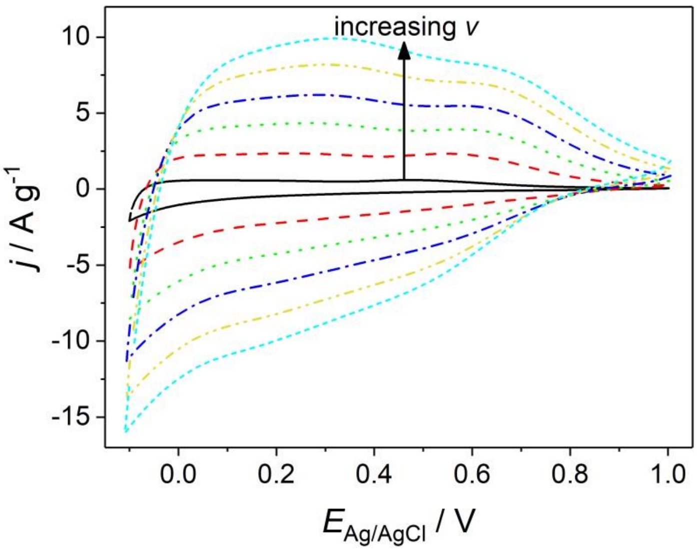

Figure S3: Cyclic voltammograms recorded at different scan rates for a $ZnMnO_3$/ZnO composite electrode obtained by photodeposition for 45 min. Electrolyte: 1 M Na_2SO_4 aqueous solution purged with N_2; scan rates: v = 0.005 V s^{-1}; 0.020 V s^{-1}; 0.040 V s^{-1}; 0.060 V s^{-1}; 0.080 V s^{-1}; 0.100 V s^{-1}.

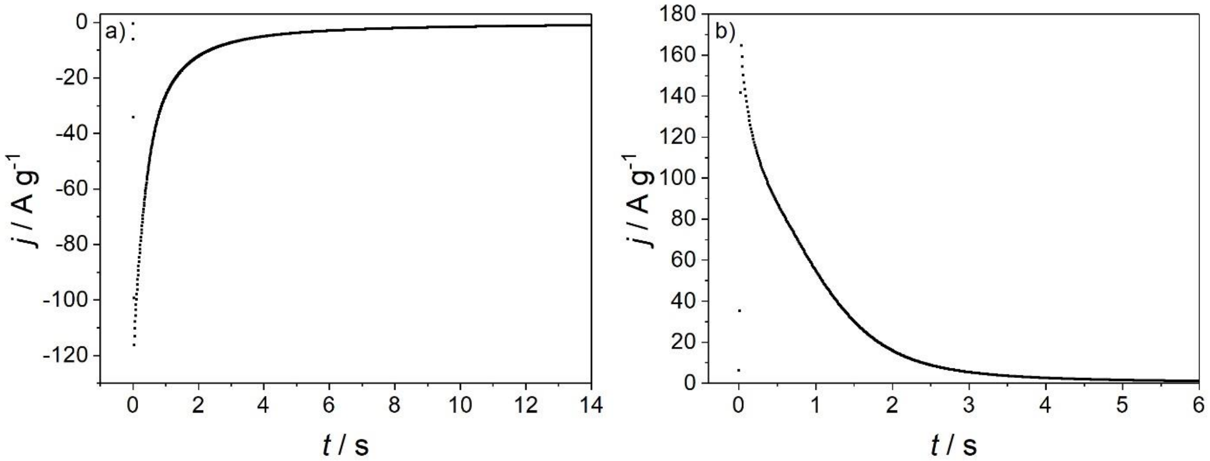

Figure S4: Current transients recorded for a $ZnMnO_3$/ZnO electrode upon electron accumulation (a) and extraction (b). The current transients were measured upon stepping the potential from $E_{Ag/AgCl}$ = 1.0 V to $E_{Ag/AgCl}$ = -0.1 V (charging) and back to $E_{Ag/AgCl}$ = 1.0 V (discharging). Each potential was applied for t = 150 s. Electrolyte: 1 M Na_2SO_4 aqueous solution purged with N_2.

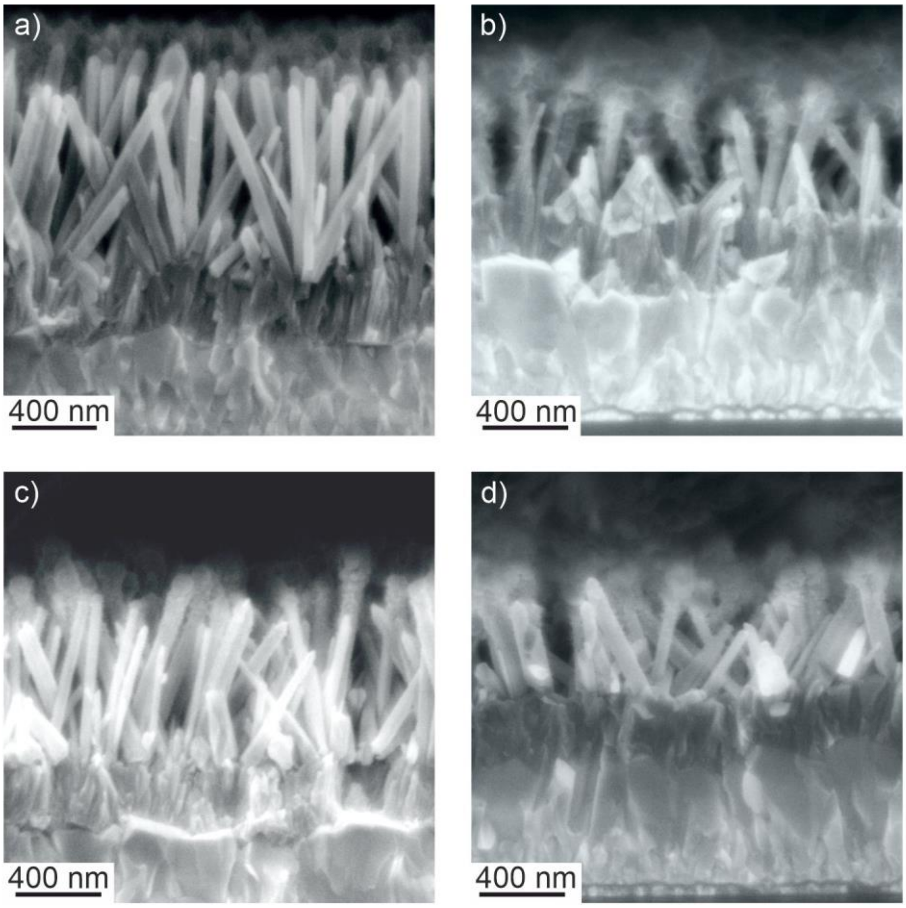

Figure S5: Cross section scanning electron micrographs of a ZnO nanowire electrode before photodeposition (a) and of $ZnMnO_3$/ZnO composite electrodes after 45 min of electrodeposition at pH 4 (b), pH 7 (c) and pH 10 (d).

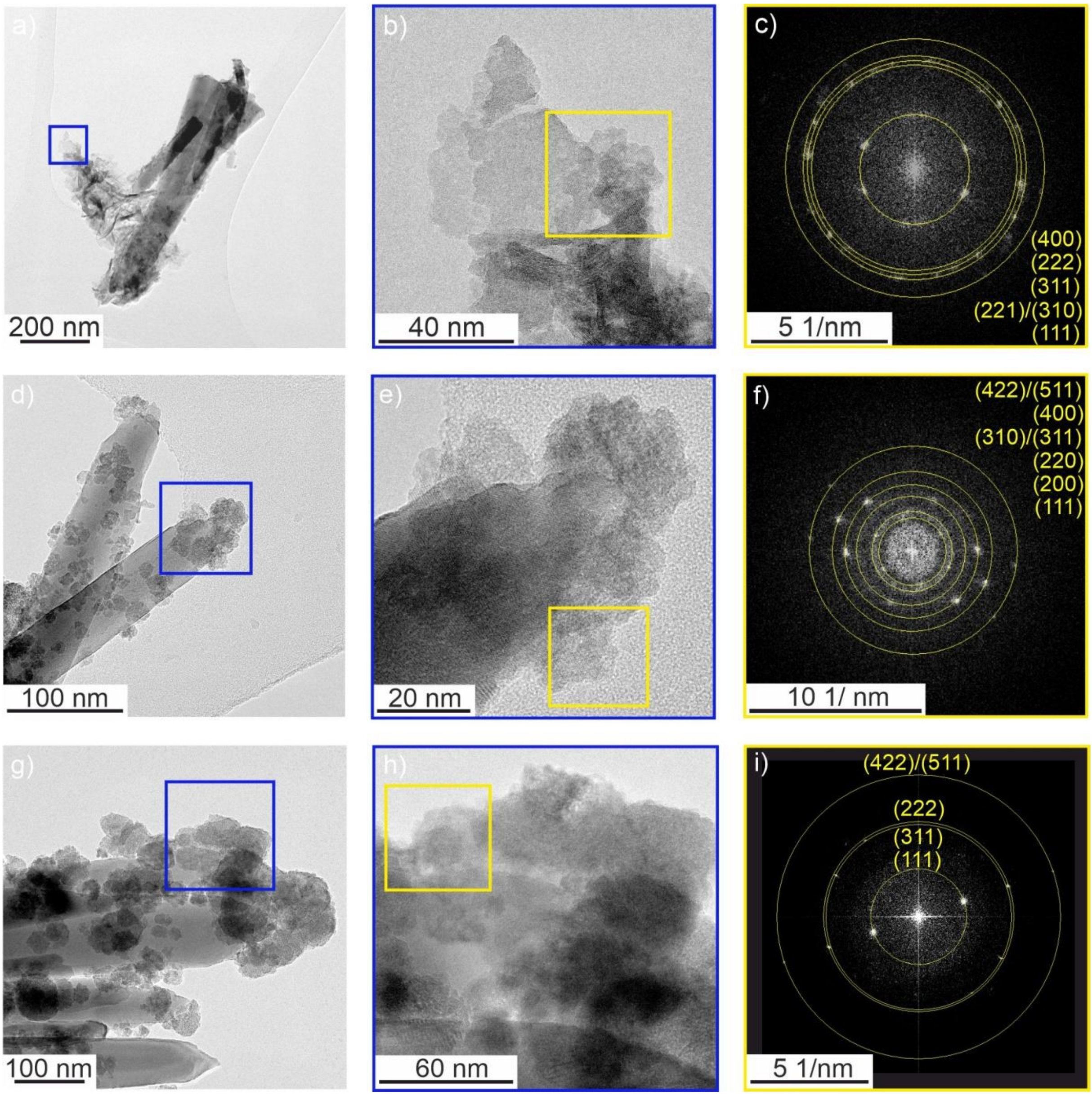

Figure S6: (a,d,g) Transmission electron micrographs and (b,e,h) high resolution TEM images (corresponding to sample regions indicated by blue squares in (a),(d) and (g), respectively) of ZnO nanowires following electrodeposition (deposition time: 45 min) at pH 4 (a,b,c), pH 7 (d,e,f) and pH 10 (g,h,i). (c,f,i) FFT patterns of sample spots indicated by yellow and red squares in (b), (e) and (h), respectively. FFT spots are assigned to lattice planes in defective cubic spinel $ZnMnO_3$ and organized from low to high real space lattice spacings (see also Table S2).

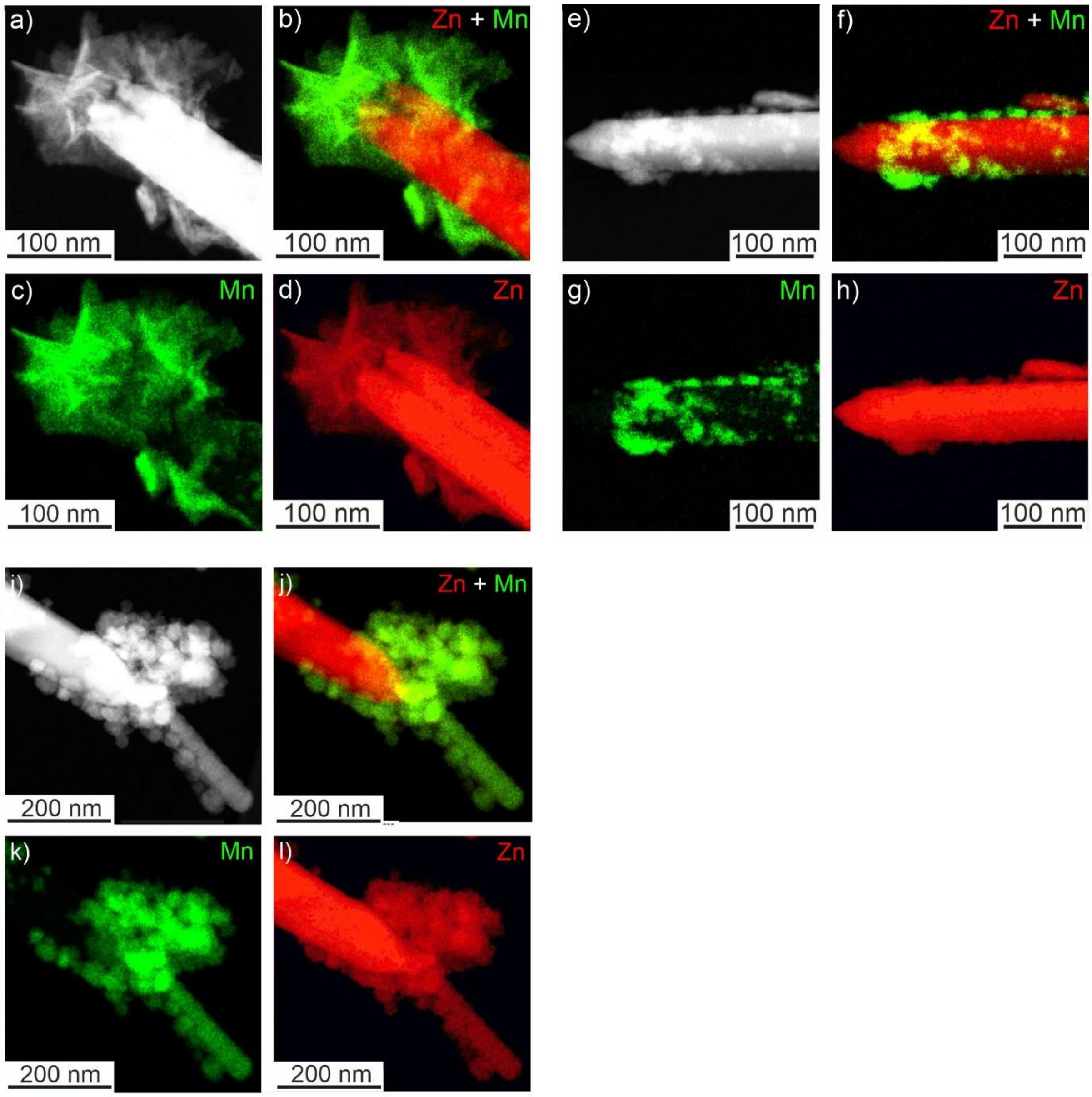

Figure S7: STEM-HAADF image (a,e,i) and elemental intensity maps (b-d, f-h, j-l) as obtained by EDX analysis of composite nanostructures resulting from electrodeposition (deposition time: 45 min) at pH 4 (a-d), pH 7 (e-h) and pH 10 (i-l). Single elemental maps of Mn (c,g,k) and Zn (d,h,l) are combined to a mixed elemental map (b,f,j).

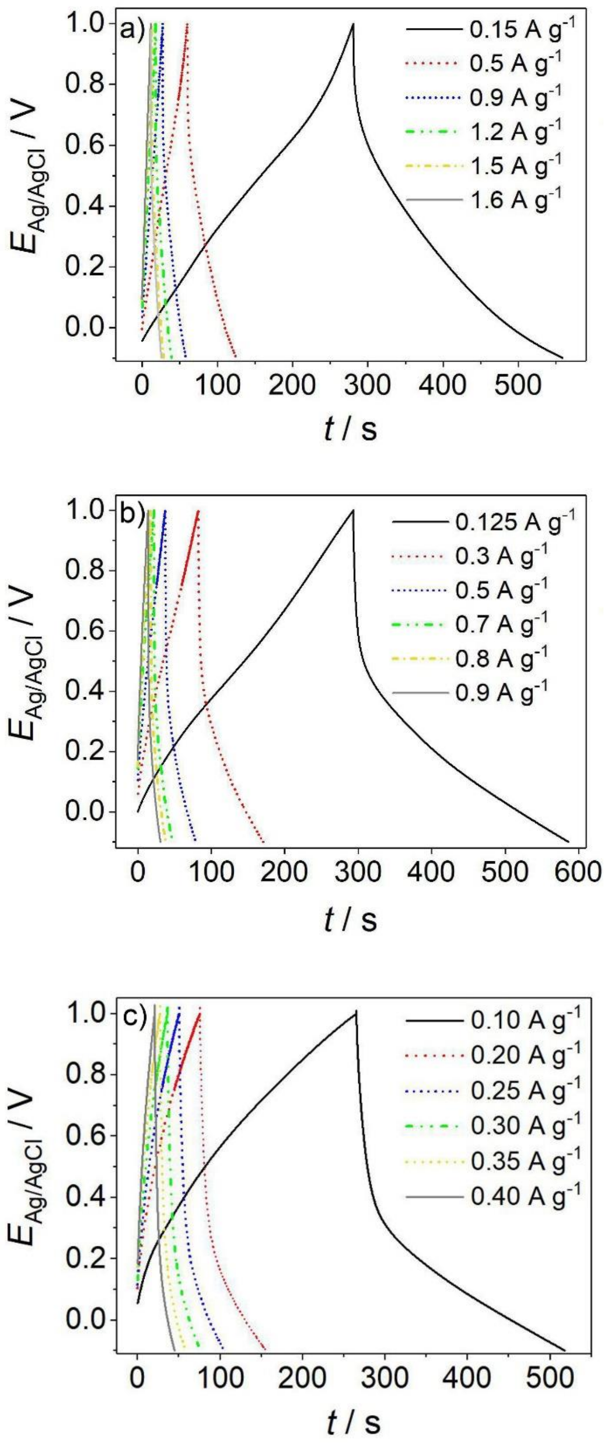

Figure S8: (a) Galvanostatic charging-discharging curves recorded at different current densities for $ZnMnO_3$/ZnO composite electrodes after 45 min of electrodeposition at pH 4 (a), pH 7 (b) and pH 10 (c). Electrolyte: 1 M Na_2SO_4 aqueous solution purged with N_2.

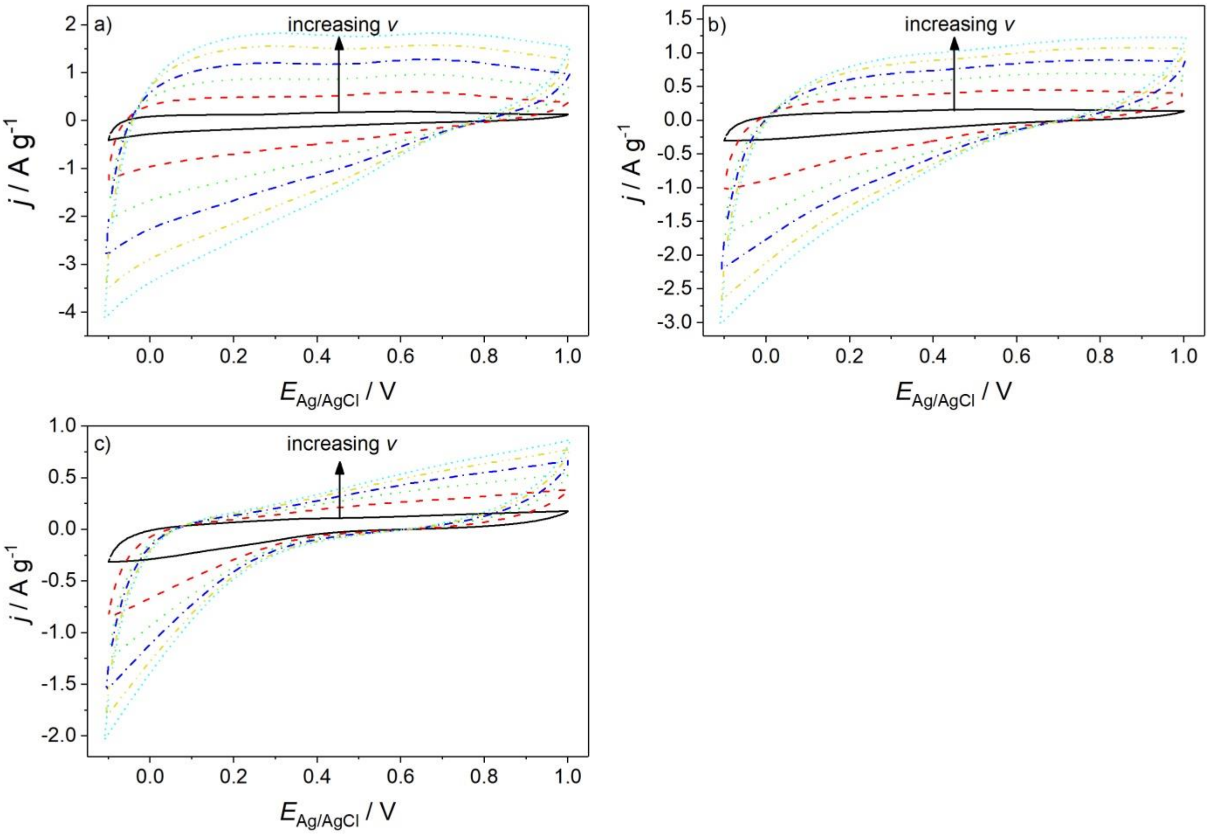

Figure S9: Cyclic voltammograms recorded at different scan rates for $ZnMnO_3$/ZnO composite electrodes electrodeposited at pH 4 (a), pH 7 (b) and pH 10 (c). Electrolyte: 1 M Na_2SO_4 aqueous solution purged with N_2; scan rates: v = 0.005 V s^{-1}; 0.020 V s^{-1}; 0.040 V s^{-1}; 0.060 V s^{-1}; 0.080 V s^{-1}; 0.100 V s^{-1}.

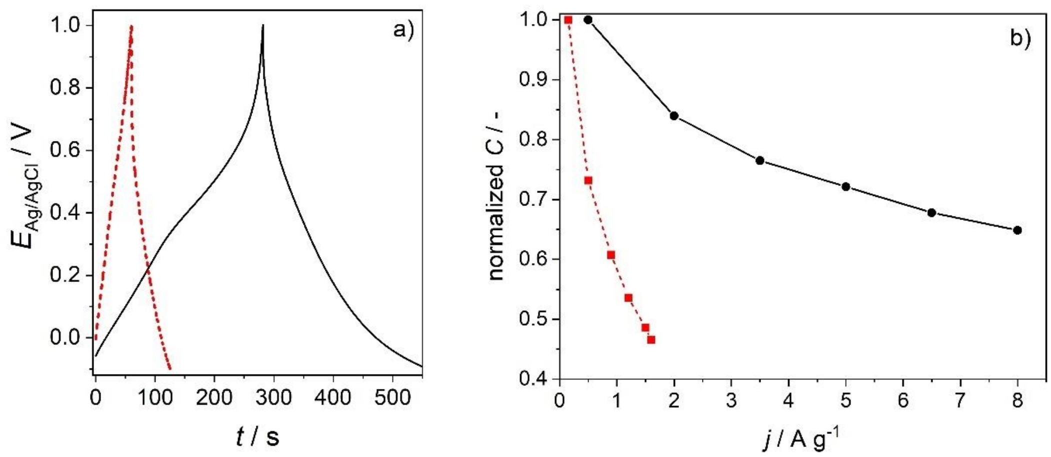

Figure S10: (a) Galvanostatic charging-discharging curves at j = 0.500 A g^{-1} and (b) normalized specific capacitance as a function of current density (as estimated from data in Figures S8 and 7a) for $ZnMnO_3$/ZnO composite electrodes from electrodeposition at pH 4 (red dotted lines) and photodeposition (black solid lines), respectively. The specific capacitance values determined at the lowest current density were normalized to one to allow for a comparison of the rate capability. Specific capacitance (as referenced to the total electrode mass i.e. the mass of ZnO nanowires and of the $ZnMnO_3$ deposit): electrodeposited film: C = 36 F g^{-1} at j = 0.150 A g^{-1}; photodeposited film: C = 130 F g^{-1} at j = 0.5 A g^{-1}; Electrolyte: 1 M Na_2SO_4 aqueous solution purged with N_2.

Supplementary Tables

Table S1: Comparison of experimentally determined values of lattice spacings $d_{exp.}$ (as extracted from FFT patterns, Figure S2) with literature values $d_{Lit.}$ in defective cubic spinel $ZnMnO_3$.[5]

$d_{exp.}$ / Å	$d_{Lit.}$ / Å	lattice plane	phase
1.5 ± 0.1	1.4105	(531)	$ZnMnO_3$
	1.4752	(440)	
	1.6060	(511)	
2.5 ± 0.1	2.4089	(222)	$ZnMnO_3$
	2.5161	(311)	

Table S2: Comparison of experimentally determined values of lattice spacings $d_{exp.}$ (as extracted from FFT patterns, Figure S6) with literature values $d_{Lit.}$ in defective cubic spinel $ZnMnO_3$.[5]

	**$d_{exp.}$ / Å**	**$d_{Lit.}$ / Å**	**lattice plane**	**phase**
Figure S6a-c	4.9 ± 0.1	4.8179	(111)	$ZnMnO_3$
	2.7 ± 0.1	2.7816/2.6388	(221)/(310)	
	2.5 ± 0.1	2.5161	(311)	
	2.4 ± 0.1	2.4089	(222)	
	2.1 ± 0.1	2.0862	(400)	
Figure S6d-f	4.9 ± 0.1	4.8179	(111)	$ZnMnO_3$
	4.2 ± 0.1	4.1724	(200)	
	3.0 ± 0.1	2.9503	(220)	
	2.5 ± 0.1	2.5161	(311)	
	2.1 ± 0.1	2.0862	(400)	
	1.6 ± 0.1	1.7034/1.6060	(422)/(511)	
Figure S6g-i	4.9 ± 0.1	4.8179	(111)	$ZnMnO_3$
	2.5 ± 0.1	2.5161	(311)	
	2.4 ± 0.1	2.4089	(222)	
	1.6 ± 0.1	1.7034/1.6060	(422)/(511)	

References

[1] R. Tena-Zaera, J. Elias, G. Wang, C. Lévy-Clément, *J. Phys. Chem. C* **2007**, *111*, 16706–16711.

[2] R. Tena-Zaera, J. Elias, C. Lévy-Clément, I. Mora-Seró, Y. Luo, J. Bisquert, *Phys. Status Solidi A* **2008**, *205*, 2345–2350.

[3] K. Rettenmaier, G.A. Zickler, G.J. Redhammer, T. Berger, *ChemPhysChem* **2023**, *24*, e202200586.

[4] C.-C. Hu, C.-C. Wang, *Electrochem. Commun.* **2002**, *4*, 554–559.

[5] L.V. Saraf, P. Nachimuthu, M.H. Engelhard, D.R. Baer, *J. Sol-Gel Sci. Technol.* **2010**, *53*, 141–147.
